# Who Will Use Pre-Exposure Prophylaxis (PrEP) and Why?: Understanding PrEP Awareness and Acceptability amongst Men Who Have Sex with Men in the UK – A Mixed Methods Study

**DOI:** 10.1371/journal.pone.0151385

**Published:** 2016-04-19

**Authors:** Jamie Frankis, Ingrid Young, Paul Flowers, Lisa McDaid

**Affiliations:** 1 School of Health and Life Sciences, Glasgow Caledonian University, Glasgow, United Kingdom; 2 MRC/CSO Social and Public Health Sciences Unit, University of Glasgow, Glasgow, United Kingdom; University of Pittsburgh, UNITED STATES

## Abstract

**Background:**

Recent clinical trials suggest that pre-exposure prophylaxis (PrEP) may reduce HIV transmission by up to 86% for men who have sex with men (MSM), whilst relatively high levels of PrEP acceptability have been reported to date. This study examines PrEP awareness amongst sub-groups of MSM communities and acceptability amongst MSM in a low prevalence region (Scotland, UK), using a mixed methods design.

**Methods:**

Quantitative surveys of n = 690 MSM recruited online via social and sociosexual media were analysed using descriptive statistics and multivariate logistic regression. In addition, n = 10 in-depth qualitative interviews with MSM were analysed thematically.

**Results:**

Under one third (29.7%) of MSM had heard of PrEP, with awareness related to living in large cities, degree level education, commercial gay scene use and reporting an HIV test in the last year. Just under half of participants (47.8%) were likely to use PrEP if it were available but there was no relationship between PrEP acceptability and previous PrEP awareness. Younger men (18–25 years) and those who report higher risk UAI were significantly more likely to say they would use PrEP. Qualitative data described specific PrEP scenarios, illustrating how risk, patterns of sexual practice and social relationships could affect motivation for and nature of PrEP use.

**Conclusion:**

These findings suggest substantial interest PrEP amongst MSM reporting HIV risk behaviours in Scotland. Given the Proud results, there is a strong case to investigate PrEP implementation within the UK. However, it appears that disparities in awareness have already emerged along traditional indicators of inequality. Our research identifies the need for comprehensive support when PrEP is introduced, including a key online component, to ensure equity of awareness across diverse MSM communities (e.g. by geography, education, gay scene use and HIV proximity), as well as to responding to the diverse informational and sexual health needs of all MSM communities.

## Introduction

Pre-exposure prophylaxis (PrEP) is the use of antiretroviral medication by HIV negative individuals to reduce the risk of HIV transmission. Findings from iPrEx [1] and other PrEP trials [2] led the US Food and Drug Administration to approve Truvada^®^ (emtricitabine/tenofovir disoproxil fumarate) for use as PrEP in July 2012 [3]. However, to date, PrEP has not yet been regulated for clinical use in other countries and uptake of PrEP in the US outside of trial settings is limited [4]. A recent Lancet editorial [5] describes access to PrEP as an issue of equity and human rights, calling on global health organizations, national health policy makers and pharmaceutical manufacturers to increase and expand PrEP access across low, middle and high income countries. Such large scale implementation requires a better understanding of PrEP acceptability amongst potential users.

In Scotland, just under half (47%) of new HIV infections occurred amongst men who have sex with men (MSM) in 2014 [6]. MSM are disproportionately affected by HIV infection globally [7], and are generally seen as the key subgroup for PrEP intervention in high-income countries [8–10]. Recent British [8] and French [9] trials have demonstrated that PrEP can reduce HIV transmission amongst MSM by up to 86%. Reviews of PrEP acceptability research amongst MSM found that whilst PrEP awareness was consistently low (14%-25%), PrEP acceptability (predominantly assessed through likelihood, or willingness, of use) varied considerably (28%-80%; most over 50%) [11,12]. Although multiple demographic and psycho-social factors have been associated with willingness to use PrEP, including lower levels of education, younger age and reporting UAI, no strong similarities emerged across these studies [11,12]. Within the UK, surveys of MSM found that reported HIV-risk behaviour and regular testing/sexual health clinic attendance were significant factors in PrEP acceptability [10,13].

Given the relatively high levels of PrEP acceptability reported to date, there is a need to better understand which sub-groups within MSM communities are willing to use PrEP and why. The aim of the current study is to explore PrEP awareness and acceptability amongst MSM in a low prevalence region (Scotland, UK), combining qualitative and quantitative data. Scotland has stable HIV prevalence [14] and an on-going yet stable undiagnosed fraction, which suggests continued incidence [15]. Our comprehensive methodological approach capitalizes upon the strengths of diverse data sets to produce a holistic and nuanced understanding of which subgroups of MSM are most likely to accept PrEP and to address the complexities of PrEP use in real life; a critical step in planning effective PrEP implementation.

## Materials and Methods

### Study Design

This paper employs a mixed methods design, drawing on two datasets–a cross-sectional survey and one-to-one semi-structured interviews—which were sequentially collected and concurrently analysed.

### Survey Data

The SMMASH (Social Media, MSM and Sexual Health) (see S1 Fig for a copy) survey collected anonymous, self-complete questionnaires from men recruited online via gay-specific sociosexual media websites (Gaydar, Recon and Squirt) and smartphone apps (Grindr and Gaydar) and Facebook from November 2012 to February 2013. These sociosexual media websites/apps differ from social media in that they offer primarily sexually, rather than socially, oriented online interactions. All members of these sociosexual media, which have a minimum user age of 18, were invited to participate. Facebook adverts targeted only male users aged over 18 who ‘liked’ various gay-related phrases inviting them to participate. Full details of the survey were provided to participants on the landing webpage, highlighting they were under no obligation to take part and participation taken as evidence of informed consent. No financial participation incentive was given. Participants were asked not to complete the questionnaire if they had already done so but duplicates were not screened for. Fraudulent and partial entries were screened and deleted.

In total, 1326 men in Scotland completed useable questionnaires. Questionnaires surveyed demographics (age, residence, education, proximity to and frequency of gay scene use), sexual health (HIV/STI testing and diagnoses) and sexual behaviour in the previous 12 months. A measure of unprotected anal intercourse (UAI) with higher risk for HIV infection was created to include men who reported UAI with ≥2, casual, and/or HIV status unknown/discordant partners in the previous 12 months (compared with men reporting UAI with 0/1, regular and/or HIV status known/concordant partners only). These measures were used previously in the MRC/CSO Gay Bar Studies [15].

A brief description of PrEP was provided: *“A drug (called Truvada) has been licensed in America to reduce the risk of sexually acquiring HIV for people who are HIV negative*. *This is known as Pre-Exposure Prophylaxis (PrEP—prophylaxis just means ‘prevention’)*. *In order for the drug to work properly*, *it needs to be taken once a day and never missed*. It can reduce the chance of HIV infection for men who have sex with men by 73%* if taken every day. *It doesn’t have any serious side effects but it can cause nausea in the first month for about 10% of people who take it*. *The drug is not yet available in the UK*.” (*Note, This figure of 73% was accurate at the time of data collection [1]). PrEP awareness was assessed by asking whether participants had previously heard of PrEP (Response: Yes, No, Unsure). Answers were recoded into two categories: ‘unsure / had not heard of PrEP’ vs ‘heard of PrEP’. PrEP acceptability was assessed by asking participants about their likelihood of PrEP use if it were available today, assessed on a 7 point Likert Scale from extremely likely to extremely unlikely, subsequently recoded into 3 categories; likely, unsure and unlikely to create a simpler variable with more equal group sizes. Development of these PrEP awareness and acceptability questions was informed by a recent systematic review of PrEP awareness and acceptability studies [11]. Ethical approval was granted by Glasgow Caledonian University School of Health and Life Sciences Ethics Subcommittee (HLS id: B11/59) and consent assumed by survey participation.

### Qualitative Data

We conducted in-depth interviews (IDIs) with communities affected by HIV in the UK (MSM and migrant African men and women) as part of a wider qualitative study exploring the acceptability of PrEP and Treatment as Prevention (TasP) in Scotland. Inclusion criteria for interview participants was to be 18 years or older, live in Scotland and to identify as either gay, bisexual or be a man who has sex with men (any ethnicity) or to be a man or a woman born in Africa but living in Scotland. For the purposes of this analysis, we draw on a interview data with HIV-negative and/or untested MSM participants. Participants were recruited through community, health and social media channels between March and September 2013. Exploration of the acceptability of PrEP included discussions concerning awareness, potential use, concerns and combination with existing risk management strategies. IDIs were digitally recorded, transcribed verbatim and transcripts were anonymised to ensure participant confidentiality. Written consent was provided by all participants and ethical approval was obtained from the University of Glasgow’s College of Social Sciences Ethics Committee (CSS2012/0193, CSS20120264). Further details on methods, including explanatory descriptions and interview schedule can be found elsewhere [16].

### Data Analyses

The qualitative and quantitative data sets were analysed concurrently. Quantitative data were analysed with IBM SPSS 21 by the first author. Men who were HIV positive (n = 75) were excluded from this analysis as were men with missing data on any of the regression variables (n = 561) leaving a sample size of 690 participants. This included 599 (86.8%) men who reported they were HIV-negative and 91 (13.2%) who were untested/unsure of their HIV status. Chi-square tests were used for bivariate comparisons. Variables significant at the bivariate level (p<0.05) were entered into two logistic regression models used to estimate odds ratios (OR) and 95% confidence intervals (CI) for PrEP awareness and acceptability. T-test and Chi^2^ analyses were used to compare the demographic and behavioural differences between participants included in the analysis (n = 690) with those excluded due to missing data (n = 561).

Simultaneously, qualitative data were analysed thematically drawing on both anticipated and emergent themes [17,18]. Once a full set of themes were established, anonymised transcripts were added to and coded in Nvivo 10, a qualitative data software management package. Themes were justified and consistently applied using analytical memorandum. Transparency of coding was established through use of NVivo 10. Rigour throughout the analysis was achieved through an iterative process of discussion and revision between co-authors [17,19].

#### Integrating the findings

Following separate data analyses, the findings were combined. For the purposes of this paper, we identified relevant themes from the qualitative analysis that further explained or provided more nuance to the quantitative results. In particular, we looked specifically at themes from the qualitative analysis in relation to awareness of PrEP and likelihood of PrEP use, and drew on these to provide illustrative examples of how PrEP might be used and in what context.

## Results

### Participants

#### Survey sample

The characteristics of the survey sample are shown in Table 1. The mean age of men sampled was 37 years (range 18–84, SD = 12.9), 97.7% were white and whilst most (82.3%) were gay identified, one in six were bisexual (16.8%) and several identified as straight (0.9%). Over half (59.6%) were single but 31.9% had a regular male partner or civil partner (necessarily same-sex, in current UK law) and 8.6% had a regular female partner or were married to a woman. Over two-thirds had degree or postgraduate level education. Participants were drawn almost evenly from Glasgow (31.3%) and Edinburgh (26.4%), Scotland’s two largest cities, with a further 42.3% from the rest of the country. Most men infrequently used the commercial gay scene (defined herein as gay bars, clubs and saunas), leading us to collapse this into a bivariate measure of either Never (n = 273; 39.6%) or Ever (n = 417, 60.4%). Just under half said that their nearest commercial gay scene was not within easy reach. One third reported higher risk UAI, just under half reported an STI test (43.9%) or an HIV test (46.8%), and just under 1 in 10 reported a positive STI test in the previous 12 months.

**Table 1:** Survey Sample Characteristics (N = 690).

	n	%
**Age**		
18–25	164	23.8
26–35	181	26.2
36–45	143	20.7
46+	202	29.3
**Ethnicity**		
White	670	97.7
Black	2	0.3
Asian	5	0.7
Mixed / other	9	1.3
**Sexual Orientation**		
Gay	568	82.3
Bisexual	116	16.8
Straight	6	0.9
**Relationship Status**		
Single	411	59.6
Regular Male Partner / Civil Partnership	220	31.9
Regular Female Partner / Married to a woman	59	8.6
**Education**		
Secondary	83	12.0
Further	141	20.4
Degree	466	67.5
**Postcode**		
Glasgow	216	31.3
Edinburgh	182	26.4
Rest of Scotland	292	42.3
**Gay scene use**		
Never	273	39.6
Once a month or less	263	38.1
At least twice a month	154	22.3
**Proximity to gay scene**		
Too far	318	46.1
Near	372	53.9
**Higher risk UAI in the previous 12 months**		
Yes	233	33.8
No	457	66.2
**STI Test in the previous 12 months**		
Yes	303	43.9
No	387	56.1
**STI in the previous 12 months**		
Yes	65	9.4
No	625	90.6
**HIV test in the previous 12 months**		
Yes	323	46.8
No	367	53.2
**Previously heard of PrEP**		
Yes	205	29.7
No	465	67.4
Unsure	20	2.9
**Likelihood of using PrEP if it were available today**		
Likely	330	47.8
Uncertain	171	24.8
Unlikely	189	27.4

#### Missing data analysis

Men who were excluded from this analysis due to missing data were significantly more likely to report a bisexual or straight identity (Chi^2^ = 4.62, p = 0.032), never use the commercial gay scene (chi2 = 5.24, p = 0.022), report no high risk UAI (Chi^2^ = 11.91, p = 0.001), no STI (Chi^2^ = 4.67, p = 0.027) or HIV test (Chi^2^ = 8.01, p = 0.005) test in the last year. However, they were no more likely to have heard of PrEP (Chi^2^ = 0.87, ns) nor report they were likely to use PrEP (Chi^2^ = 0.05), p = 0.829)

#### Qualitative sample

We draw on IDI data from a larger qualitative study of communities affected by HIV in Scotland (n = 34). The sample for this combined analysis includes 10 HIV negative/status unknown MSM aged between 19 and 52 years (median 27.5 years), resident across four urban and semi-urban Scottish regions. Participants identified as gay (n = 9) or bisexual (n = 1), white (n = 9) and British (n = 7). We did not collect information on income or education levels as part of the qualitative study.

### Understanding PrEP awareness amongst MSM in Scotland

Two-hundred and five men (29.7%) had heard of PrEP, 20 (2.9%) were unsure and 465 (67.4%) had not (see Table 1). Table 2 shows the factors associated with participants’ awareness of PrEP. When controlling for the factors significant at the bivariate level in the multivariate logistic regression, the adjusted odds of having heard of PrEP remained significantly higher for men living in Glasgow (AOR = 2.1) and Edinburgh (AOR = 1.94) compared to the rest of Scotland, men who had degree level education (AOR = 3.68) compared to secondary level education, men who ever used the commercial gay scene (AOR = 1.49) and men who reported an HIV test in the previous 12 months (AOR = 2.25). However, PrEP awareness was not related to higher risk UAI in the multivariate model.

**Table 2:** Factors associated with Awareness of PREP (N = 690).

	Heard of PrEP (n = 205, 29.7%), n (%)	Unaware of PrEP (n = 485, 70.3%), n (%)	Bivariate Regression Analyses			Multivariate Regression Analyses		
**Age**			**OR**	**95% CI**	**p value**	**AOR**	**95% CI**	**p value**
18–25	41 (20)	123 (25.4)	1		0.246			
26–35	60 (29.3)	121 (24.9)	1.49	(0.93–2.38)	0.098			
36–45	48 (23.4)	95 (19.6)	1.52	(0.92–2.49)	0.100			
46+	56 (27.3)	146 (30.1)	1.15	(0.72–1.84)	0.557			
**Postcode**								
Rest of Scotland	60 (29.3)	232 (47.8)	1		<0.001			0.002
Glasgow	79 (38.5)	137 (28.2)	2.23	(1.5–3.32)	<0.001	2.1	(1.34–3.27)	0.001
Edinburgh	66 (32.2)	116 (23.9)	2.2	(1.45–3.33)	<0.001	1.94	(1.22–3.08)	0.005
**Sexual Orientation**								
Bisexual / Straight	26 (12.7)	96 (19.8)	1					
Gay	179 (87.3)	389 (80.2)	1.7	(1.06–2.71)	0.026			
**Relationship Status**								
Regular female ptr	9 (4.3)	50 (10.3)	1		0.038			
Single	124 (60.5)	287 (59.2)	2.4	(1.45–5.03)	0.020			
Regular male ptr	72 (35.1)	148 (30.5)	2.7	(1.26–5.8)	0.011			
**Education**								
Secondary	11 (5.4)	72 (14.8)	1		<0.001	1		<0.001
Further	32 (15.6)	109 (22.5)	1.92	(0.91–4.06)	0.087	2.08	(0.96–4.52)	0.058
Degree	162 (79)	304 (62.7)	3.49	(1.8–6.77)	<0.001	3.68	(1.85–7.32)	<0.001
**Gay scene use**								
Never	62 (30.2)	211 (43.5)	1			1		
Ever	143 (69.8)	274 (56.5)	1.78	(1.25–2.52)	0.001	1.49	(1.02–2.17)	0.039
**Proximity to gay scene**								
Far	76 (37.1)	242 (49.9)	1					
Near	129 (62.9)	243 (50.1)	1.69	(1.21–2.36)	0.002			
Had any higher risk UAI*								
No	125 (61)	332 (68.5)	1					
Yes	80 (39)	153 (31.5)	1.39	(0.99–1.95)	0.058			
**Talked about HIV with UAI partners**								
Never	25 (12.2)	78 (16.1)	1		0.013			
No UAI	99 (48.3)	270 (55.7)	1.14	(0.69–1.9)	0.602			
Always/Sometimes	81 (39.5)	137 (28.2)	1.85	(1.09–3.13)	0.023			
**Had an STI Test in the previous 12 months**								
No	93 (45.4)	294 (60.6)	1					
Yes	112 (54.6)	191 (39.4)	1.85	(1.33–2.58)	<0.001			
**Had an STI in the previous 12 months**								
No	180 (87.8)	445 (91.8)	1					
Yes	25 (12.2)	40 (8.2)	1.55	(0.91–2.62)	0.107			
**Had an HIV test in the previous 12 months**								
No	79 (38.5)	288 (59.4)	1					
Yes	126 (61.5)	197 (40.6)	2.33	(1.67–3.26)	<0.001	2.25	(1.28–3.95)	0.005

OR = odds ratio; AOR = adjusted odds-ratio; 95% CI = 95% confidence interval.

*UAI with 2 or more partners, UAI with casual partners, and/or UAI with unknown/discordant partners in the previous 12 months.

Illuminating these findings further, qualitative analysis suggested that PrEP awareness was also affected by proximity to HIV. Of the few participants who had heard of PrEP, most were likely to be HIV-positive, have friends who were, or who worked in field of sexual health.

*“Talking to my friend at [a sexual health charity in England]*…*He started talking to me a lot about the prevention pill*. *This is getting them all quite excited and*, *you know*, *they’re talking a lot about that”*

*(IDI*, *46+ years)*.

### Understanding the likelihood of PrEP use

Half of the survey participants (47.8%) reported being likely to use PrEP if it were available today, whilst one quarter (24.8%) were uncertain and one quarter (27.4%) unlikely to use it (see Table 1). Table 3 shows the factors associated with participants’ likelihood of PrEP use if it were available today, comparing men who were ‘likely’ to use PrEP (47.8%) with those who were ‘unlikely/unsure’ (52.2%). When controlling for the factors significant at the bivariate level in the multivariate logistic regression, the adjusted odds of the likelihood of PrEP use remained significantly higher for men aged 18–25 compared to men in the three older age groups (25–35 AOR = 0.61; 36–45 AOR = 0.47; 46+ AOR = 0.54) and for men who reported any higher risk UAI in the previous 12 months (AOR = 2.27). Finally, awareness of PrEP was not associated with the likelihood of PrEP use.

**Table 3:** Factors associated with the acceptability of PrEP use (N = 690).

	Willing to use PrEP (n = 330, 47.8%), n (%)	Unsure/ Unlikely to take PrEP (n = 360, 52.2%), n (%)	Bivariate Regression Analyses			Multivariate Regression Analyses		
**Age**			**OR**	**95% CI**	**p value**	**AOR**	**95% CI**	**p value**
18–25	97 (29.4)	67 (18.6)	1		0.008	1		0.007
25–35	85 (25.8)	96 (26.7)	0.61	(0.4–0.94)	0.024	0.61	(0.4–0.95)	0.028
36–45	60 (18.2)	83 (23.1)	0.5	(0.32–0.79)	0.003	0.47	(0.30–0.75)	0.001
46+	88 (26.7)	114 (31.7)	0.53	(0.35–0.81)	0.003	0.54	(0.35–0.82)	0.004
**Postcode**								
Rest of Scotland	138 (41.8)	154 (42.8)	1		0.967			
Glasgow	104 (31.5)	112 (31.1)	1.04	(0.72–1.47)	0.843			
Edinburgh	88 (26.7)	94 (26.1)	1.05	(0.72–1.51)	0.817			
**Sexual Orientation**								
Bisexual/Straight	64 (19.4)	58 (16.1)	1					
Gay	266 (80.6)	302 (83.9)	0.8	(0.54–1.18)	0.259			
**Relationship Status**								
Regular female ptr	31 (9.4)	28 (7.8)	1		0.511			
Single	200 (60.6)	211 (58.6)	0.86	(0.5–1.48)	0.577			
Regular male ptr	99 (30)	121 (33.6)	0.74	(0.42–1.31)	0.303			
**Education**								
Secondary	41 (12.2)	42 (11.7)	1		0.954			
Further	67 (20.3)	74 (20.6)	0.93	(0.54–1.60)	0.786			
Degree	222 (67.3)	244 (67.8)	0.93	(0.58–1.49)	0.768			
**Gay scene use**								
Never	124 (37.6)	149 (41.4)	1					
Ever	206 (62.4)	211 (58.6)	1.17	(0.86–1.59)	0.306			
**Proximity to gay scene**								
Far	146 (44.2)	172 (47.8)	1					
Near	184 (55.8)	188 (52.2)	1.15	(0.85–1.56)	0.352			
Had any higher risk UAI*								
No	193 (58.5)	264 (73.3)	1			1		
Yes	137 (41.5)	96 (26.7)	1.95	(1.42–2.69)	<0.001	2.27	(1.37–3.78)	0.002
**Talked about HIV with UAI partners**								
Never	58 (17.6)	45 (12.5)	1		0.015			
No UAI	158 (47.9)	211 (58.6)	0.58	(0.37–0.90)	0.016			
Always/Sometimes	114 (34.5)	104 (28.9)	0.85	(0.53–1.36)	0.501			
**Had an STI Test in the previous 12 months**								
No	178 (53.9)	209 (58.1)	1					
Yes	152 (46.1)	151 (41.9)	1.18	(0.88–1.6)	0.277			
**Had an STI in the previous 12 months**								
No	294 (89.1)	331 (91.6)	1					
Yes	36 (10.9)	29 (8.1)	1.4	(0.84–2.34)	0.201			
**Had an HIV test in the previous 12 months**								
No	170 (51.5)	197 (54.7)	1					
Yes	160 (48.5)	163 (45.3)	1.14	(0.84–1.54)	0.399			
**Heard of PrEP**								
No / Unsure	231 (70)	254 (70.6)	1					
Yes	99 (30)	106 (29.4)	1.03	(0.74–1.42)	0.873			

OR = odds ratio; AOR = adjusted odds-ratio; 95% CI = 95% confidence interval.

*UAI with 2 or more partners, UAI with casual partners, and/or UAI with unknown/discordant partners in the previous 12 months

Qualitative data analyses enhance these population level findings by suggesting motivation for potential PrEP use. Participants described the specific scenarios in which they would consider using PrEP, illustrating how the social complexities of risk and sex could be critical factors in decisions about potential use. Motivation was often identified in relation to the use or non-use of condoms. For example, PrEP could be seen as a ‘back-up’ option for men who would regularly use condoms.

*“I perceive myself as a condom user with occasional lapses*. *I’d have thought that’s the sort of person who would benefit most from PrEP if it works”*

*(IDI*, *46+ years)*.

Similarly, some men identified PrEP as ideal for situations where regular patterns of sexual practice, and related condom use, might be disrupted, such as holidays or in the case of alcohol and/or drug use.

*“If I was aware of [PrEP] before and … I was going on holidays or something like that*, *I think I would make sure that I would have … enough for when I’ve been away or if I was at any risk*, *I think I would definitely avail of [PrEP] and make sure that I had it in place*. *But again personally*, *if I was having a few drinks and I knew I was having [PrEP] I probably still wouldn’t comply with the condoms*, *my foolishness in the past [is evidence] of that”*

*(IDI*, *18–25 years)*.

Motivation for PrEP use was also identified in relation to sexual partners and the nature of sexual relationships. Some men suggested they might specifically use PrEP in a sexual relationship with a known HIV-positive sexual partner.

*“I mean if that was available*, *and say I was going out with somebody that was HIV positive*. *I would take that*, *and—without a doubt I would take that*. *I’ve no concern whatever for the sake of swallowing one pill a day”*

*(HIV-negative MSM*, *IDI*, *18–25 years)*.

In contrast, others felt that PrEP was only a short-term solution to HIV prevention, and therefore, something they would use for casual sexual encounters with partners of unknown sero-status.

*“I would definitely consider it more of an outside [of] relationship thing and if I was being*, *going back to the word*, *promiscuous I think I would definitely*, *yeah*, *I would*, *I would feel much more reassured knowing that that was there*. *It’s kind of like a wee bit further on from PEP [Pre-Exposure Prophylaxis]”*

*(IDI*, *18–25 years)*.

In summary, whilst quantitative data suggested that only younger age and reporting higher risk UAI increased men’s likelihood of using PrEP, qualitative data further illustrated how the complex interplay of risk perception, patterns of sexual practice and social relationships could influence the motivation for and nature of PrEP use.

## Discussion

This is the first mixed methods study to consider awareness and acceptability of PrEP in the UK. Amongst MSM in Scotland, awareness of PrEP was associated with living in large cities, higher levels of educational attainment, using the commercial gay scene and taking an HIV test within the last year. In terms of understanding who would use PrEP, once made aware of it, younger men and those reporting higher risk UAI within the previous year were most likely to say they would use it. Qualitative analysis demonstrated how motivation was tied to patterns of condom use and sexual relationships (e.g. with known HIV positive partners, as an emergency back-up system and episodic rather than continuous use). These findings suggest that there is substantial interest in biomedical HIV risk reduction strategies amongst MSM reporting HIV risk behaviours in Scotland and, given the efficacy shown by the Proud [8] and Ipergay [9] trials, a case to investigate mainstream PrEP implementation within the UK.

Overall, less than one third (29.7%) of MSM recruited online in Scotland had heard of PrEP, similar to Scottish men recruited on the commercial gay scene [10] and MSM in other high income countries (11–23%; [11]; 14–25%; [12,20]). Moreover, our findings suggest structural factors pattern who currently is aware of PrEP, including greater urbanicity, higher educational qualifications, sexual health literacy (as reflected by recent HIV testing), HIV proximity and embeddedness within gay communities. Although these associations resonated with previous studies, little consistency in the relationship between PrEP awareness, sociodemographic and behavioural variables has been shown [12] even amongst MSM within Scotland [10]. Despite little PrEP engagement from health promotion agencies worldwide, it appears that disparities in awareness have already emerged along traditional indicators of inequality. Clearly, a challenge to PrEP implementation is to promote PrEP to men who are not reached through work within traditional MSM locations such as large cities, the commercial gay scene and STI clinics. Since almost 80% of our online survey participants rarely or never use the commercial gay scene, digitally mediated social and gay sociosexual networks offer one a promising solution. Online campaigns can be geographically targeted to consumers outwith large urban centres and would also provide access to younger MSM, who use sociosexual networks more often than older men [21]. As such, a broad, multimedia social marketing campaign, encapsulating both traditional (commercial gay scene and STI clinics) and digital (social and sociosexual media) marketing designed to reach MSM is warranted. Crucially, in order for PrEP to be targeted appropriately and used effectively, widening PrEP awareness is a necessary first step.

Almost half of participants (47.8%) reported that they would use PrEP if it were available today. This high level of interest in PrEP is similar to research with Scottish men recruited on the commercial gay scene [10] as well as multiple studies conducted with MSM elsewhere [11,12]. Amongst our respondents, younger men and those who report higher risk UAI were significantly more willing to adopt PrEP. Multiple studies corroborate this relationship between younger age [10,13,22–24], UAI [10,13,23–28] and PrEP acceptability, with relatively few studies reporting no age based such relationship [29] or significant alternative demographic or behavioural relationships [10]. This level of interest amongst men reporting higher-risk UAI in our study suggests that PrEP could be an acceptable prevention method to those currently at risk of HIV, echoing previous findings [12,29]. Moreover, since both the Proud [8] and Ipergay [9] trials suggested that PrEP efficacy is somewhat higher than the 73% suggested in our questionnaire, and that intermittent use is equally effective, our PrEP acceptability rate is likely an under-estimate.

Finally, it is important to highlight that awareness of PrEP was *not* related to PrEP acceptability. This reinforces the notion that information alone is insufficient to support PrEP usage and underlines the need for both awareness-raising and support for PrEP use to enable cost-effective targeting. In particular, those motivating factors identified in our research, including the role of PrEP in diverse sexual relationships, and episodic or ‘emergency’ cases, will need to be addressed therein.

### Limitations

Our novel combination of quantitative and qualitative data allowed us to examine both population level findings and in-depth subjective understandings. It was not possible to calculate a response rate for the quantitative data given the nature of online surveys, and men’s use of multiple sites/profiles. However, the sample size achieved herein is comparable to other surveys of MSM in Scotland [15] and other comparable countries [30]. It is also clear that our findings rely on men’s estimates of their future behaviour, rather than any objective measure of actual behaviour. Nonetheless, the current absence of PrEP availability means that such estimates represent the only population level evidence to inform subsequent PrEP provision. Missing data were high for variables in our final regression analyses and significant demographic and behavioural differences between those men excluded and included were apparent which limit the validity of our results. The small sample size of the study’s qualitative component, and recruitment of some participants who were already engaged in sexual health services, limit the generalisability of these qualitative findings to a wider population of MSM. However, we would argue they are transferrable to similar populations and help to further elucidate the population level findings. The key HIV behavioural risk measure adopted herein is limited in scope, since by dichotomising risk it elides more nuanced analysis of degrees of risk. Equally, it blurs the distinction between numbers of unprotected partners and unprotected acts, the latter of which is a more accurate risk indicator. Furthermore, it restricts risk estimates to a fixed time span of the previous 12 months. Although behavioural risks over a longer period are relevant in terms of HIV risk at the individual level, retrospective estimates of population behaviour are likely to become increasingly inaccurate as the recall period increases. Finally, whilst it should be noted that there are often differences between anticipated and actual behaviour–the ‘intention-behavioural gap’ often discussed in health psychology [31], rather than representing predictive research, our analysis attempts to indicate potential PrEP interest and identify those issues to be considered in relation to future PrEP implementation. Indeed, we would argue that potential PrEP use is underpinned by a complex interplay of social context, risk management strategies and relationship status, alongside demographic characteristics and behavioural intentions.

### Implications

Awareness of PrEP remains modest amongst MSM in Scotland, though willingness to use PrEP is relatively high. Two key groups emerged as most likely to be willing to adopt PrEP; younger MSM and MSM who report higher risk UAI in the previous year. This is encouraging since the latter group are those very men most likely to benefit from PrEP use. Mixed responses to PrEP in conjunction with condom use indicate a range of combination prevention PrEP strategies will be employed by potential PrEP users. Some men viewed PrEP as incompatible with condom use, whilst others felt it provided a ‘safety net’ in terms of additional prevention. These findings have important policy implications for HIV prevention in the UK, as well as other similar low prevalence, high income and geographically dispersed contexts, such as Australia, New Zealand and Canada. Our research identifies the need for comprehensive support, including a key online component, when PrEP is introduced to ensure equity of awareness across diverse MSM communities (e.g. by geography, education levels, gay scene use and HIV proximity), as well as to respond to the diverse informational and sexual health needs of all MSM communities.

It is vital to consider PrEP amidst the increasing complexity of HIV prevention, and intersectionality with diverse risk management strategies (e.g. seroadaptive behaviours). Critically, within the context of correct PrEP use, *condomless* anal intercourse no longer represents a higher risk behaviour for HIV infection, but instead is part of a safer sexual strategy. As such, ‘high risk’ men might incorporate new strategies as described herein (e.g. episodic PrEP, either with positive partners or as a back-up) which changes the risk in specific sexual acts and may explain their interest in PrEP [23,29]. Certainly, MSM who came forwards to participate in the UK PROUD study could be categorised as ‘very high HIV risk’ in terms of their UAI behaviour [32]. Moreover, recent discussions have focused on this episodic PrEP strategy during periods of risk [33] as described by our participants. This highlights the importance of considering multiple PrEP strategies beyond daily, continuous usage. Guidance which embraces episodic and situation based PrEP use and ‘prevention-effective adherence’ [33], alongside the sustained PrEP use model currently envisaged, will more closely meet MSM’s own behavioural expectations. Moreover, such guidance must relate to the contextual and temporal specifics of PrEP implementation amongst those who need it most. Crucially, such complex risk management strategies, including PrEP, TasP, regular HIV testing and condom use require high levels of HIV literacy throughout the MSM population in order to effectively reduce HIV incidence.

## Supporting Information

S1 Fig The SMMASH Survey. Note: this fig. contains only those questions from the SMMASH Survey included within the current paper. The full SMMASH survey is available from the corresponding author.
(PDF)
