# Supplementary material for: Who Will Use Pre-Exposure Prophylaxis (PrEP) and Why?: Understanding PrEP Awareness and Acceptability amongst Men Who Have Sex with Men in the UK – A Mixed Methods Study
Source: PLoS One. 2016 Apr 19;11(4):e0151385. doi: 10.1371/journal.pone.0151385 (PMC4836740; doi:10.1371/journal.pone.0151385)
Supplement: S1 Fig — Note: this fig. contains only those questions from the SMMASH Survey included within the current paper. The full SMMASH survey is available from the corresponding author. (PDF) [file pone.0151385.s001.pdf]

Men who have Sex with Men in Scotland Social & Sexual Networking Survey 201...

Information Sheet

What's this all about?

We want to look at how you use Internet and mobile phone sites like Facebook, Gaydar and Recon to network with your friends and meet guys for sex. So we're conducting a survey.

Who should take part?

Men who have sex with men (or want to), are over 18 years old, live in Scotland and use any social networking websites (e.g. Facebook, Gaydar, Recon etc.) or social networking phone apps (e.g. Grindr, etc.).

Who is conducting this survey and who funds it?

Researchers at Glasgow Caledonian University are working together with three sexual health organisations (Gay Men's Health, the Terrence Higgins Trust and Waverley Care). These institutions are funding this study. Gaydar.co.uk have kindly donated their mail shot to all of their members in Scotland for free to promote this survey!

Why carry out this survey?

We want to provide appropriate online health advice and future services for men in Scotland who have sex with men. In order to do this, we need to find out which sites and 'apps' you use, how you use them and what you use them for. To plan the content of those services and to understand how socio-sexual networking contributes to health, we need to explore your sexual and sexual health behaviours.

What is involved?

The survey will only take about 15 minutes. You should complete it only once. It is entirely voluntary, anonymous and completely confidential. We do not store, or even know, your email or IP address. We're using SurveyMonkey to collect the data. We've set it up so there is no way the completed questionnaire can be traced back to you. You are free to leave out any questions you do not want to answer, or which you think are not relevant, although we hope you will answer all that you can. Ethical approval for this study has been granted by Glasgow Caledonian University. You do not need to take part in the survey if you do not wish to.

Is it secure?

SurveyMonkey are a well respected online survey company. It's illegal for them to use the

information you provide or pass it on to 3rd parties. All data are transferred using encrypted Internet protocols and kept in a secure location. As an online participant in this research, there is always the risk of intrusion by outside agents (i.e. hacking). However, SurveyMonkey take security really seriously and do as much as possible to ensure this won't happen.

What will be done with my survey answers?

Your responses will be added to the answers of everyone else and entered onto a computer for statistical analysis. We keep these data securely according to the Data Protection Act and will delete them when we've finished our work. We aim to use the information you give us to devise new strategies to improve the sexual health of men who have sex with men in Scotland and to publish academic papers in journals to share our knowledge with others interested in the sexual health of men. However, it is important that you understand that taking part in this study may have no direct benefit to you.

If you've already filled in this survey online in the last few weeks, thanks so much for your help, but please don't do it again – once is enough.

Many thanks for your time and help.

If you have any questions or comments about this survey, please email Jamie Frankis at Glasgow Caledonian University at j.frankis@gcu.ac.uk

If you'd like any information on sexual health and safer sex including where to get a named HIV test before you complete the survey, please Click Here.

INFORMED CONSENT: Please select your choice below.

Clicking on the "next" button below indicates that:

• you have read the above information

• you voluntarily agree to participate

• you are at least 18 years of age

If you do not wish to participate in the research study now or at any point during the survey, please decline participation by clicking on the 'withrdaw from survey' button in the top right hand corner.

About You

1. What age are you?

[ ]

2. What is the first part of your postcode? (e.g. G42)

[ ]

3. Please tell us what your highest educational qualification is? (e.g. Standard Grades, Highers, A Levels, Degree)

[ ]

4. Sexual Orientation (please choose one)

- ○ Gay
- ○ Bisexual
- ○ Straight

Other (please specify)

[ ]

5. Ethnicity (please choose one)

- ○ White Scottish
- ○ White British
- ○ White Irish
- ○ Any other white background
- ○ Pakistani
- ○ Indian
- ○ Bangladeshi or any other East Asian
- ○ Chinese
- ○ Other South Asian
- ○ African
- ○ Caribbean, Black Scottish or any other Black background
- ○ Any Mixed background
- ○ Any other background

Other (please specify)

[ ]

6. What is your current partnership status? (please tick all that apply)

- ☐ Single
- ☐ Boyfriend / Regular male partner
- ☐ Civil Partnership / Married to a man
- ☐ Girlfriend / Regular female partner / Married to a woman

Other (please specify)

[ ]

About your sexual behaviours

We'd now like to find out about your sexual behaviours in the last year.

These questions are completely confidential and anonymous.

Remember you can miss out any questions you don't want to answer.

7. With how many men have you had <u>any sexual contact</u> in the last 12 months?

[ ]

8. With how many men have you had <u>anal sex</u> in the last 12 months?

[ ]

9. Have you had <u>anal sex WITHOUT a condom</u> with a man in the last 12 months?

- ○ Yes
- ○ No

Anal sex without a condom in the last 12 months

We'd like to ask you more about the men you had anal sex WITHOUT a condom with in the last 12 months.

10. With how many men have you had <u>anal sex WITHOUT a condom</u> in the last 12 months?

[ ]

Thinking about the times you had anal sex WITHOUT a condom in the last 12 months…

11. How often was this with a casual partner? (please tick one)

- ○ Always
- ○ Sometimes
- ○ Never

12. How often did you talk about HIV with your partners? (please tick one)

- ○ Always
- ○ Sometimes
- ○ Never

13. Were any of these partners HIV positive? (please tick one)

- ○ Yes, all
- ○ Yes, some
- ○ No
- ○ Don't know

HIV testing

We'd now like to ask you about you HIV status and testing behaviours.

Remember these questions are confidential and anonymous.

You can also miss out any questions you do not want to answer.

14. What do you believe your current HIV status is?

- HIV positive
- HIV negative
- Don’t know

15. When was your most recent HIV test?

- In the last 6 months
- Between 6 months and 1 year ago
- Between 1 and 5 years ago
- Over 5 years ago
- Never had an HIV test

About your HIV testing

16. What was the result of your last HIV test?

- HIV positive
- HIV negative
- Don’t know

Sexual Health Testing, Condoms and Advice

17. Have you had a sexually transmitted infection (STI) in the last 12 months?

- Yes
- No

18. When was your most recent STI test?

- In the last 6 months
- Between 6 months and 12 months ago
- Over 12 months ago
- Never had an STI test

Gay Scene Use and Being 'Out'

19. In the last month, how often did you go out on the gay scene?

- 4-5 times a week
- 1-2 times a week
- 2-3 times a month
- Once a month or less
- Never

Pre-Exposure Prophylaxis (PrEP) Knowledge, Acceptability and Behaviour

A drug (called Truvada) has been licensed in America to reduce the risk of sexually acquiring HIV for people who are HIV negative. This is known as Pre-Exposure Prophylaxis (PrEP - prophylaxis just means ‘prevention’). In order for the drug to work properly, it needs to be taken once a day and never missed. It can reduce the chance of HIV infection for men who have sex with men by 73% if taken every day. It doesn’t have any serious side effects but it can cause nausea in the first month for about 10% of people who take it. The drug is not yet available in the UK.

20. Before this survey had you ever heard about Pre-Exposure Prophylaxis, or what we have been calling PrEP, that can be used to prevent HIV infection?

- Yes
- No
- Don't know

21. If this PrEP pill were available today, how likely would you be to use it? (choose one)

- Extremely Unlikely
- Very Unlikely
- Somewhat Unlikely
- Not Sure
- Somewhat Likely
- Very Likely
- Extremely Likely

Pre-Exposure Prophylaxis (PrEP) Knowledge, Acceptability and Behaviour

Thank-you!

Thanks for all your time and help with this project!

The success of this project, and our ability to use it to improve sexual health services for men who have sex with men in Scotland really depends on people like **you** taking the time to help us. We really appreciate this.

Study Withdrawal

Now that you've seen all of the questions, it is important that we give you the opportunity to withdraw from the study.

If you want to withdraw from this survey, please click the button on the top right hand side. **This will automatically withdraw you from this survey and delete all of the answers you've given so far from our database instantly - *you won't be asked to confirm this*.** No record that you took this survey will be kept.

If you're happy to submit your answers, please click 'next'.

Advice and Support

Sexual Health Advice and Support

This questionnaire has covered some really sensitive issues about your sexual behaviours and sexal health.

If you'd like to find out more information about sexual health, find out how to get tests for sexually transmitted infections (including HIV) or talk to someone about this all, completely confidentially please **CLICK HERE** (opens in a new window) or go to **http://www.hiv-wakeup.org.uk/.**

Alternatively you can use the HIV Scotland Services finder to locate HIV testing and other sexual health services near where you live.

Services for people living with HIV are provided by the Terrence Higgins Trust and Waverley Care.

Waverley Care also provide services for people living with Hepatitis C.

General information about sexually transmitted infections for men who have sex with men is provided by Gay Men's Health.

Counselling is also available in Scotland for gay and bisexual men and people who are affected by HIV or Hepatitis C.

If you're done, click 'Done' to submit your answers and finish this survey.

Thank-you you once again,

Jamie Frankis, Gaydar.co.uk and the Social Netreach Network team

Glasgow Caledonian University

GAYDAR.CO.UK
WHAT YOU WANT, WHEN YOU WANT IT

SNN SCOTTISH NETREACH NETWORK

Terrence Higgins Trust

GAY MEN'S HEALTH gmh

Waverley Care
making a positive difference
